# Tracing a Route and Finding a Shortcut: The Working Memory, Motivational, and Personality Factors Involved

**DOI:** 10.3389/fnhum.2018.00225

**Published:** 2018-05-30

**Authors:** Francesca Pazzaglia, Chiara Meneghetti, Lucia Ronconi

**Affiliations:** ^1^Department of General Psychology, University of Padova, Padova, Italy; ^2^Department of Philosophy, Sociology, Pedagogy, and Applied Psychology, University of Padova, Padova, Italy

**Keywords:** virtual exploration, wayfinding, visuospatial working memory, mental rotation, personality traits, pleasure in exploring, spatial anxiety, self-efficacy

## Abstract

Wayfinding (WF) is the ability to move around efficiently and find the way from a starting point to a destination. It is a component of spatial navigation, a coordinate and goal-directed movement of one’s self through the environment. In the present study, the relationship between WF tasks (route tracing and shortcut finding) and individual factors were explored with the hypothesis that WF tasks would be predicted by different types of cognitive, affective, motivational variables, and personality factors. A group of 116 university students (88 F.) were conducted along a route in a virtual environment and then asked first to trace the same route again, and then to find a shortcut between the start and end points. Several instruments assessing visuospatial working memory, mental rotation ability, self-efficacy, spatial anxiety, positive attitude to exploring, and personality traits were administered. The results showed that a latent spatial ability factor (measured with the visuospatial working memory and mental rotations tests) – controlled for gender – predicted route-tracing performance, while self-report measures of anxiety, efficacy, and pleasure in exploring, and some personality traits were more likely to predict shortcut-finding performance. We concluded that both personality and cognitive abilities affect WF performance, but differently, depending on the requirements of the task.

## Introduction

### Wayfinding: Multiple Abilities and Processes

Wayfinding (WF) is generally defined as the ability to move around efficiently and find the way from a starting point to a destination (Montello, 2005).

It is widely acknowledged that WF is a multicomponent ability (Hegarty et al., 2006; Wolbers and Hegarty, 2010) susceptible to broad individual differences (Hegarty and Waller, 2005), ranging from individuals who suffer from severe orientation deficits from childhood onward (Iaria et al., 2009; Iaria and Burles, 2016; Piccardi et al., 2017) to people with excellent orientation skills (Verde et al., 2015). It has been largely established that various mechanisms and processes are implicated in WF (Wolbers and Hegarty, 2010), and served by a complex network of neural substrates (Wegman et al., 2014).

The direct involvement of different working memory (WM) components has been demonstrated by using dual task paradigms in experiments in which participants were asked to perform WF tasks while concurrently performing secondary tasks assumed to load different WM components (Garden et al., 2002; Labate et al., 2014; Meilinger et al., 2008). On the whole, these studies proved that visuospatial working memory (VSWM) is implicated in the performance of spatial navigation tasks. These results converge with those of other studies using structural equation modeling that found a role for VSWM in the performance of spatial navigation tasks (Allen et al., 1996; Meneghetti et al., 2016). Correlational and structural equation modeling studies likewise found relationships between scores obtained in tests on spatial abilities [mainly involving mental rotation tasks (MRT)] and in WF tasks (Hegarty et al., 2006; Muffato et al., 2017).

There is also evidence of stress and/or anxiety harming WF performance (Hund and Minarik, 2006; Walkowiak et al., 2015), particularly in difficult tasks (Srinivas, 2011). Spatial anxiety, i.e., the degree of anxiety experienced when performing spatial tasks, is related to a worse performance in navigation tasks (e.g., Lawton, 1994, 2010; Schmitz, 1997). It may act as a mediator in gender-related differences, and be associated with particular spatial representation strategies (Lawton and Kallai, 2002). Schmitz (1997) found that spatial anxiety slowed WF performance in a virtual environment (VE); and Lawton (1994) showed that it correlated with route WF strategy use, and that more spatial anxiety was associated with less spatial competence.

As for the positive emotions, it was found that individuals who take pleasure in exploring places tend to have a good sense of direction (De Beni et al., 2014), and perform better in spatial tasks (Meneghetti et al., 2014; Muffato et al., 2016, 2017). In the same vein, Pazzaglia et al. (2017) showed that a significant part of the variability in the performance of a shortcut-finding task was explained by an aggregate measure of pleasure in exploring and spatial self-efficacy. Interestingly, the strength of the relationship between subjective measures and WF tasks seems to depend on how difficult the task is: the tougher the task, the stronger the relationship (Weisberg et al., 2014; Pazzaglia et al., 2017)

The study by Pazzaglia et al. (2017) suggests that self-efficacy, as well as anxiety, may affect WF behavior. Bandura (1997) described self-efficacy (a motivational factor traditionally defined from a socio-cognitive perspective) as a person’s belief in their ability to accomplish a task. Its influence has been demonstrated in a number of domains, including: cognitive development (Bandura, 1993); self-regulated learning and academic motivation (Schunk and Di Benedetto, 2014); and performance in sports (Moritz et al., 2000).

Other factors relating to spatial task performance have been explored from a socio-cognitive perspective too. For instance, stereotype threat (Maass and Cadinu, 2003), and gender identification (Yang and Merrill, 2016) revealed a role in determining performance in mental rotation tasks: young women did worse in the MRT when under stereotype threat than in a non-stereotyped control condition (Moè and Pazzaglia, 2006); and gender identification seemed to interact with stereotype threat in worsening MRT performance (Nori et al., 2009). Taken together, the literature reviewed above suggests that emotions and motivation can play a part in spatial learning, and these factors need to be further explored in the specific case of WF.

Another order of variables that might influence WF ability regards personality. Already Tolman (1938) introduced personality variables among the factors prone to affect navigation behavior in rats. Bryant (1982) subsequently found that personality measures correlated with self-reports of Sense of Direction (SOD, flexibility, worrying about becoming lost, dominance, capacity for status, sociability, social presence, self-acceptance, well-being, and intellectual efficiency), and pointing errors (capacity for status, sociability, social presence, and self-acceptance), and concluded that personality dispositions are important to the acquisition and accuracy of mental representations of the environment.

In line with these assumptions the literature supports a relationship between certain personality traits and performance in spatial and WF tasks. Extroversion is one of the personality traits most often studied, and findings indicate that extroverts are more likely to have an exploratory behavior (Wyllie and Smith, 1996), and to score higher for self-reported SOD (Condon et al., 2015), a measure that predicts performance in environment tasks (Hegarty et al., 2006). Wyllie and Smith (1996) found that adolescents scoring high in extroversion were more likely to explore the environment and spend their leisure time in places farther from home than their less extrovert counterparts. Condon et al. (2015) found a correlation between scores for extroversion and self-reported SOD, and also with other personality traits, such as conscientiousness, intellect, and emotional stability.

More recently, Walkowiak et al. (2015) explored the relationship between three major personality traits and the time taken, the mistakes made, and the length of the path covered in a WF task in a VE, which involved retracing a route just learned. They found moderate correlations between psychoticism (i.e., less emotional stability) and the variables considered, high scores for psychoticism being associated with a worse spatial performance. They explained these results as being due to participants scoring high on psychoticism being more erratic and exploratory in their WF. The same study revealed moderate correlations between WF variables and WF anxiety, as measured on the Wayfinding Anxiety Scale (Lawton and Kallai, 2002). Another study supporting a relationship between personality traits and self-reported WF competence was conducted by Yang and Merrill (2016), who found that more feminine personality characteristics (described, among others, as being affectionate and gentle) predicted a worse self-reported WF competence.

The above-mentioned studies generally corroborate the idea of a connection between personality and spatial competence, but some aspects remain unexplored. First of all, we need to establish more precisely which specific mechanisms link some personality traits with performance in spatial tasks, and the role of potential mediators (as discussed in Bryant, 1982). Second, personality has so far been considered mainly with reference to subjective measures of spatial navigation, such as SOD (Condon et al., 2015; Yang and Merrill, 2016), or spatial worrying (Bryant, 1982), or to spatial tasks other than navigation, such as pointing (Bryant, 1982), and mental rotation (Nori et al., 2009). Only one of the studies reviewed here analyzed the influence of personality using a WF task (Walkowiak et al., 2015). More work is needed to see whether and how certain personality traits relate to specific WF tasks. It is important to recognize that several distinctions can be drawn between different WF tasks, and they need to be taken into account in order for us to investigate the relationship between personality traits and WF ability in more depth. In this regard, it is worth noting that the general concept of WF actually involves numerous tasks that differ considerably in their features and complexity, and presumably also in the abilities required, and the cognitive processes involved.

Several attempts have been made to classify spatial navigation (Allen, 1999; Montello, 2005; Wiener et al., 2009). In empirical research, spatial learning and navigation are studied using numerous tasks and learning conditions, with distinctive implications for perception, attention, and memory. A psychologically relevant distinction is drawn between tasks that involve tracing a known route and those that entail finding a novel way to reach a destination, such as shortcut-finding tasks. The present study focuses on this distinction between route tracing and shortcut finding.

The aim of this study was to explore the influence of various individual factors on performance in two different WF tasks: route tracing and shortcut finding.

## Materials and Methods

### Participants

A total of 116 undergraduates (88 females) voluntarily took part in the study (age *M* = 21.07, *SD* = 3.97). Exclusion criteria were not adopted. All participants had adequate performances in the VSWM and MR tests, allowing us to exclude the presence of visuospatial disorders. This experiment was carried out in accordance with the recommendations of the Italian Association of Psychology (AIP) and of the Ethics Committee for Psychological Research (CERP) of University. All procedures were approved by CERP. Participants provided written consent.

### Materials

#### Pleasure in Exploring, Self-Efficacy, and Spatial Anxiety Measures

*Attitude to Spatial Exploration Questionnaire* (Attitude, revised from Pazzaglia et al., 2004) tool is designed to assess attitude to orientation tasks and pleasure in exploring. It comprises 10 statements that describe feelings, attitudes, and preferences in situations involving environmental orientation (e.g., “I love exploring different places that I still don’t know well, and finding new ways to get to places”; “I would like to play a sport like orienteering, where people have to move very fast in unknown places”). For each statement, respondents indicate their agreement on a 5-point scale from 1 (not at all) to 5 (very much), and the total score is obtained from the sum of each item rating. Internal consistency was acceptable (α = 0.68, calculated on the study sample). For the present study, we considered the total score for items 3, 6, 9, 10, which are the items specifically mentioning WF tasks (α = 0.50, calculated on the present sample). Maximum score: 20.

*Wayfinding Self-Efficacy Questionnaire* (Efficacy, revised from Mitolo et al., 2015) tool investigates how confident individuals feel about their ability to perform typical spatial tasks. It consists of 8 items that describe precise tasks (e.g., “Finding the car in a large parking lot”; “Visiting friends who live in an unfamiliar neighborhood”), scored on a 6-point scale from 1 (not at all) to 6 (very much) in response to the prompt: “Indicate how well you think you would cope in the situations described”, and the total score is given by the sum of each item rating. Internal consistency was good (α = 0.81, calculated on the present sample).

*Spatial Anxiety Questionnaire* (Anxiety, adapted from Lawton, 1994) tool investigates the levels of anxiety experienced while performing everyday spatial tasks. The items used in this scale are the same as those in the Wayfinding Self-Efficacy Questionnaire, and respondents are asked to indicate the level of anxiety experienced in the situations described. The 8 items are scored on a 6-point scale: from 1 (not at all) to 6 (very much). The final score is calculated by adding together the scores for each item. Example item: “Indicate the level of anxiety you experience in the situation described: Reaching an appointment venue in an unfamiliar part of a town.” Internal consistency was good (α = 0.82, calculated on the present sample).

In the analyses described below, for both Efficacy and Anxiety we considered the total score calculated on items 1, 2, 4, 5, 8, which refer to WF tasks in outdoor environments (Efficacy α = 0.77; Anxiety α = 0.73 calculated on the present sample). Maximum score: 30 (both for Efficacy and Anxiety scales)

#### Personality Measure

*Big Five Personality Questionnaire* (BFQ; Italian version by Caprara et al., 2008) is one of the most often used instruments for assessing personality. It comprises 134 statements that refer to 5 traits, and 2 “facets” for each trait (for a total of 10 facets, with 12 items for each facet), plus a social desirability scale measuring the respondents’ desire to give a very positive impression of themselves. For each statement, respondents indicate the extent to which they agree or disagree on a 5-point scale from 1 (very false for me) to 5 (very true for me).

The Energy trait is the level of activity, vigor, sociability, and competitiveness, in which one facet is Dynamism (activity and enthusiasm), and the other is Dominance (assertiveness and self-confidence). The Agreeableness trait refers to concern and sensitivity expressed toward others and their needs, with one facet concerning Cooperativeness (altruism and trust), and the other Politeness (kindness and civility). The Conscientiousness trait relates to self-regulation in both its proactive and its inhibitory aspects, one facet being Scrupulousness (orderliness and precision), and the other Perseverance (tenacity and persistence). The Emotional Stability trait concerns the ability to control one’s affect and emotional reactions, and one facet of this is Emotion Control (ability to handle anxiety and feelings of despondency), and the other is Impulse Control (ability to maintain control over one’s behavior). The Openness trait concerns the breadth of an individual’s cultural interests and willingness to explore and seek novelty; one facet is Openness to Culture (intellectual curiosity, interest in knowledge), and the other is Openness to Experience (interest shown toward different values and lifestyles). In a large normative population, the reliability of the five factors ranged from 0.73 to 0.90, and the reliability of the facets from 0.60 to 0.86 (Caprara et al., 2008). Maximum score for each factor: 120.

#### Visuospatial Working Memory and Spatial Ability Measures

*Corsi Blocks Task* (CBT, Corsi, 1972) is designed to test spatial WM. The apparatus used in the CBT consists of 9 identical blocks randomly placed on a board. The experimenter points to a sequence of blocks at a rate of one block per second and then asks the respondent to point at the same blocks in the same order. The length of each sequence of blocks to recall ranged from 2 to 9 blocks, and two trials were used for each sequence length. The procedure stopped when a participant was unable to reproduce both the sequences of a given length. The number of blocks in the longest sequence for which at least one of the two trials was recalled correctly was taken as the measure of the respondent’s visuospatial span. Maximum score: 8.

*Pathway Span Task* (PST, Mammarella et al., 2008) is designed to test spatial WM. Participants are asked to mentally visualize a path followed by a little man moving on a blank matrix. After the experimenter has given a set of statements regarding the directions he takes (i.e., forward, backward, to the left or right), participants are asked to indicate the man’s final position on the matrix. The complexity of the task can vary, depending on the size of the matrix (from 2 × 2 to 6 × 6) and the length of the path covered. The final score is obtained from the number of moves correctly recalled in at least two matrices out of three. Maximum score: 10.

*Mental Rotations Test* (MRT, from Vandenberg and Kuse, 1978) comprises 20 items, each consisting of one target and four alternative figures (made up of assembled cubes). The task consists in identifying which two of the four alternative figures correspond to a rotated view of the target figure. Respondents had 8 min to accomplish the task, and they scored one point when they identified both of the correct alternatives. The total score corresponded to the sum of the scores obtained for the single items. Maximum score: 20.

#### Virtual Environment

The VE was programmed in Superscape 5.61 and adapted from Pazzaglia and Taylor (2007). It consisted of a typical urban environment where we selected a specific route, some 300 meters long, comprising 12 segments and a variety of landmarks. A second VE was used for practice. The VE was presented in desktop system mode on a 17-inch screen placed 50 cm away from the participant. We opted to use a VE because it enables a greater control over the characteristics of the environment than in a real environment, and the mechanisms involved in learning a VE are much the same as in the real world (e.g., Ruddle et al., 1997; Weisberg et al., 2014).

#### Recall Tasks

Route-tracing task is involved tracing a previously learned route from a starting point to an end point, using a joystick to move forward, backward, right or left.

Shortcut-finding task is entailed using a joystick to move freely in the VE and finding the shortest path between the starting and end points of the previously learned route.

Both tasks began at the starting point used in the learning phase. The program recorded how many wrong turns were taken throughout the route in the tracing task (errors), and the length of the path covered in meters in the shortcut-finding task, which were used as dependent variables.

### Procedure

Participants were individually tested during a single session lasting about 90 min. They completed the following questionnaires in the following order: Anxiety, Attitude (pleasure in exploring), and Efficacy, plus two other questionnaires not considered in the present study. Then, the route learning phase started. Participants were told that their task was to memorize a path through a VE and then perform a number of spatial tasks. They were first familiarized with the use of the joystick and the virtual reality apparatus in a sample VE for 3 min before starting the experimental task. Participants watched an avatar walk for about 3 min from the starting point to the end point of the path. Immediately afterward, they were returned to the starting point and asked to use the joystick to trace the same route as they had just seen (route-tracing task). They were told that, if they took a wrong turn, the program would take them back to the previous intersection. If participant took three wrong turns at the same intersection, the experimenter told them which way to go (e.g., “You have to turn left here”). The program recorded how many wrong turns each participant took along the way. Then they were returned to the starting point again and asked to find the shortest way to reach the destination (shortcut-finding task). Participants were allowed to navigate the environment freely (for up to 10 min) until they reached the destination, and the route they covered was recorded. The task finished when the end point was reached, or when 10 min had passed. The dependent variables were the errors in the first task, and the length of the shortcut in the second. After the two navigation tasks, the Corsi Blocks Task and the Pathway Span Task were administered, followed by the Big Five Questionnaire, which concluded the experimental session. The order of administration of all measures (questionnaires and tasks) was the same for each participant.

## Results

### Rationale for Analyses

We conducted our analyses in four steps. First, we examined participants’ route-tracing and shortcut-finding performance to check for any broad individual differences (Hegarty and Waller, 2005). We then correlated the study variables in a second step, and used confirmatory factor analyses (CFAs) to test the relationship between the observed and latent variables in a third. This process was recommended by Schreiber (2008) to derive the best indicators of latent variables before testing a structural model. Fourth, a structural equation model (SEM) was generated using spatial ability, emotion/motivation, and personality as latent variables, and route tracing and shortcut finding as the observed variables.

Measurements and structural analyses were done using the LISREL 8.7 statistical package (Jöreskog and Sörbom, 2004). Among the various fit indexes, we adopted the root-mean-square error of approximation (RMSEA, below 0.05), the non-normed fit index (NNFI, above 0.97), the comparative fit index (CFI, above 0.97), the standardized root mean square residual (SRMR, below 0.05), and a non-significant chi-square. The issue of normality was considered too: the observed data indicated a non-significant departure from normality, as shown by Mardia’s measure of relative multivariate kurtosis (MK) obtained with the PRELIS program (Jöreskog and Sörbom, 2004): MK = 1.02 (-1.96 < *z* < 1.96).

We expected to find VSWM and spatial ability crucial to learning a path and forming a spatial mental representation of the urban environment, as suggested by a number of past studies (Garden et al., 2002; Meilinger et al., 2008; Labate et al., 2014). Once the path had been learned, however, then motivation, attitude to spatial tasks, and personality traits might be even more important to success in finding a shortcut to the same destination. Although the involvement of non-cognitive factors (personality, emotion, motivation) in WF has already been suggested (in the past studies reviewed above, for instance), our study is the first to examine a wide range of variables with reference to different WF tasks. This enabled us to see whether different groups of variables (cognitive, affective/motivational, personality) were more or less important in relation to the two tasks. Our hypotheses were tested by using path modeling, after a confirmatory factor analysis had validated our distinction of the variables in three groups: spatial ability (measured with VSWM tasks and MRT), affective/motivational factors (spatial self-efficacy, pleasure in exploring, and spatial anxiety), and several personality traits. We expected to see different patterns of relationships between the predictive variables on the one hand, and route tracing and shortcut finding on the other. Gender was inserted as an initial variable to control for its effect on all the other relationships (given its role on spatial performance; e.g., Lawton, 2010).

Based on the above-reviewed literature, we expected spatial ability to predict performance in both navigation tasks, while the affective/motivational and personality variables were expected to predict performance only in the shortcut-finding task. As for which specific personality traits might correlate with performance in the latter task, we took an exploratory stance because past research had identified different factors, from extraversion to psychoticism and dominance. Given that previous evidence showed that route tracing and shortcut finding could demand a different involvement of visuospatial competences (Labate et al., 2014; Muffato et al., 2016), the last two dependent variables were kept separate on the assumption that spatial abilities, spatial self-reports, and personality traits could affect route-tracing and shortcut-finding performance differently.

### Step 1: Individual Differences in Route-Tracing and Shortcut-Finding Performance

#### Route tracing

Although the task was quite easy (54% of participants made no or only 1 error), we found that the 25% of the sample made 5 or more errors, with a maximum of 9 errors in one case. These data confirm reports in the literature of a marked variability in the performance of navigation tasks (Hegarty and Waller, 2005).

#### Shortcut finding

**FIGURE 1:**
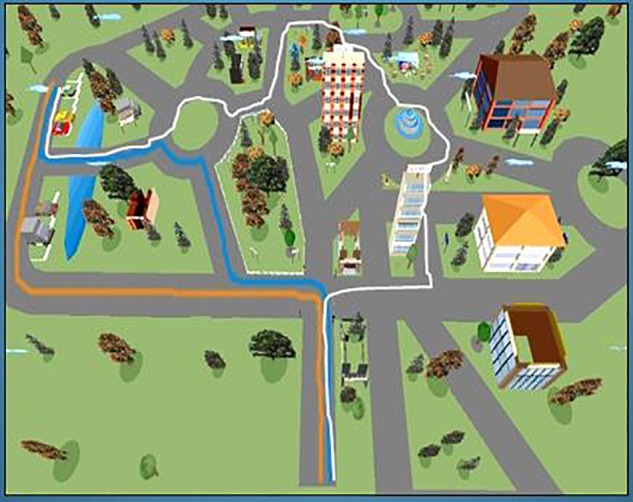
The virtual environment with the original route learned (in white) and the two shortcuts (in blue and orange) correctly identified by 50 (43%) participants.

This task revealed a broad range of individual differences too. A total of 50 of the 116 participants (43%) actually found one of the two shortest routes from the starting point to the destination (the VE, the path learned, and the two shortcuts are illustrated in **Figure 1**). Another 23 participants traced a slightly longer route, 13 covered the route they had learned previously, or one only slightly shorter (n. 11), and 18 covered much longer routes than the one they had learned, using no apparent strategy. The shortcut-finding task thus revealed individual differences that were possibly even more marked than those seen in the retracing task.

### Step 2: Correlations

**Table 1** shows descriptive statistics and correlations between all variables, revealing a distinct pattern of correlations between the measures of individual differences, and between these and the two WF tasks. As expected, the two WM tests, the Corsi Blocks Task and the Pathway Span Task, correlated moderately with each other, such as the Corsi Blocks Task and the MRT. Significantly, all these WM and spatial abilities measures showed specific correlations with the number of errors in the route-tracing task. On the other hand, the measures of pleasure in exploring, self-efficacy, and spatial anxiety correlated strongly with each other, and all showed significant correlations with the shortcut-finding task: better performance correlated with less anxiety and more self-efficacy and pleasure in exploring. Shortcut-finding performance also correlated with the personality facets Politeness and Impulse Control, referring, respectively, to the factors Agreeableness and Emotional Stability: a better performance was associated with lower scores for Politeness and Impulse Control.

**Table 1:** Correlation matrix and descriptive statistics [Means (M) and Standard Deviations (*SD*)] for the measures of interest.

	M (SD)	1	2	3	4	5	6	7	8	9	10	11	12	13	14	15	16	17	18	19
1. Gender^a^		-																		
2. Spatial anxiety	2.85 (0.80)	0.13																		
3. Pleasure in exploring	2.56 (0.64)	-0.09	-0.41***																	
4. Self-efficacy	3.74 (0.71)	-0.13	-0.60***	0.48***																
5. BFQ – Dynamism	3.38 (0.55)	0.17	-0.29**	0.22*	0.08															
6. BFQ – Dominance	3.03 (0.51)	-0.09	-0.17	0.19*	0.23*	0.37***														
7. BFQ – Cooperativeness	3.63 (0.41)	0.23*	0.15	-0.07	-0.15	0.22*	-0.20*													
8. BFQ – Politeness	3.22 (0.52)	0.09	0.09	-0.12	-0.19*	0.25**	-0.25**	0.53***												
9. BFQ – Scrupulousness	3.23 (0.66)	0.08	0.19*	-0.12	-0.02	-0.13	-0.06	0.01	-0.14											
10. BFQ – Perseverance	3.70 (0.46)	0.18	-0.03	0.22*	0.16	0.40***	0.41***	0.22*	0.08	0.24*										
11. BFQ – Emotion Control	2.63 (0.70)	-0.21*	-0.37***	0.03	0.29**	-0.03	0.01	-0.13	0.04	-0.10	-0.07									
12. BFQ – Impulse Control	2.70 (0.63)	-0.15	-0.01	-0.17	0.06	-0.27**	-0.34***	0.09	0.32***	0.01	-0.23*	0.51***								
13. BFQ – Openness to Culture	3.46 (0.46)	-0.08	-0.07	0.05	0.15	0.24**	0.18	0.11	-0.02	0.20*	0.19*	-0.18	-0.24*							
14. BFQ – Openness to Experience	3.62 (0.47)	-0.05	-0.28**	0.21*	0.25**	0.46***	0.15	0.36***	0.25**	-0.06	0.23*	-0.03	-0.06	0.46***						
15. BFQ – Lie	1.79/0.46)	0.01	-0.03	-0.08	0.02	-0.03	-0.01	-0.23*	0.01	-0.01	-0.18	0.31**	0.25**	-0.16	0.19*					
16. Mental Rotations Test	5.30 (3.84)	-0.47***	-0.16	0.02	0.16	0.05	0.04	-0.02	0.14	-0.17	-0.19*	0.18	0.13	0.06	0.10	0.11				
17. Corsi Blocks Task	6.25 (1.29)	-0.21*	-0.06	0.05	0.14	0.12	0.05	-0.07	0.02	-0.14	-0.06	0.11	0.02	-0.06	-0.04	0.12	0.32***			
18. Pathway Span Task	8.81 (1.50)	-0.05	-0.04	0.04	0.03	0.02	-0.02	-0.11	-0.06	-0.04	-0.09	-0.04	-0.10	0.04	0.12	0.11	0.14	0.21*		
19. Route-tracing task (Errors)^b^	2.05 (2.47)	0.26**	0.06	-0.04	-0.02	0.08	-0.03	0.05	0.09	0.07	0.18	-0.01	-0.01	0.04	0.06	-0.12	-0.34***	-0.30**	-0.22*	
20. Shortcut-finding task (Length)^b^	163.12 (78.92)	0.06	0.19*	-0.23*	0.22*	0.01	-0.15	0.15	0.21*	0.05	0.01	-0.01	0.19*	-0.03	0.01	0.15	-0.10	-0.01	-0.04	0.15


**N* = 116; ^∗∗∗^*p* < 0.001; ^∗∗^*p* < 0.01, ^∗^*p* < 0.05. ^*a*^The negative value of the correlations between Route tracing and Shortcut finding had a positive meaning, i.e., fewer errors and shorter lengths were associated with greater accuracy in objective measures or higher scores in subjective measures (except for spatial anxiety, where higher values had a negative meaning). ^*b*^Gender was a dichotomous variable (1 = male, 2 = female).*

### Step 3: Confirmatory Factor Analyses

#### Factor compositions

**FIGURE 2:**
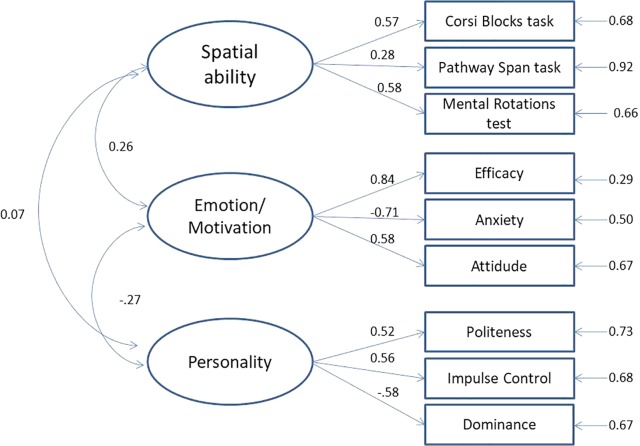
Measurement model including spatial ability, emotion/motivation self-reports, and facets of personality traits. The values reported are standardized β, all significant (*z* = 2.33 *p* < 0.05 to *z* = 7.28 *p* < 0.001) except for the correlation between spatial ability and facets of personality traits (*z* = 0.29).

In the light of previous evidence to indicate that spatial abilities constitute a single factor grouping mental rotation and VSWM (Allen et al., 1996; Hegarty et al., 2006), and that they can be distinguished by self-reported spatial measures (Hegarty et al., 2006), we tested the existence of two latent factors: spatial abilities (using the Corsi Blocks Task, the Pathway Span Task, and the MRT), and motivation/emotion (anxiety, pleasure in exploring, and self-efficacy). We also identified a third personality latent factor consisting of Politeness, Impulse Control, and Dominance, which – within each personality factor – were the facets showing the strongest correlation with the shortcut-finding task (see **Table 1**). This measurement model, based on the three factors of interest, showed good fit indices, χ^2^ = 24.19, *df* = 24 *p* = 0.45, *CFI* = 1.00, *NNFI* = 1.00, *SRMR* = 0.05, *RMSEA* = 0.008. The standardized β values are shown in **Figure 2**. The three latent variables, i.e., spatial ability, motivation/emotion, and personality, were retained in the subsequent analyses.

### Step 4: Structural Models

**FIGURE 3:**
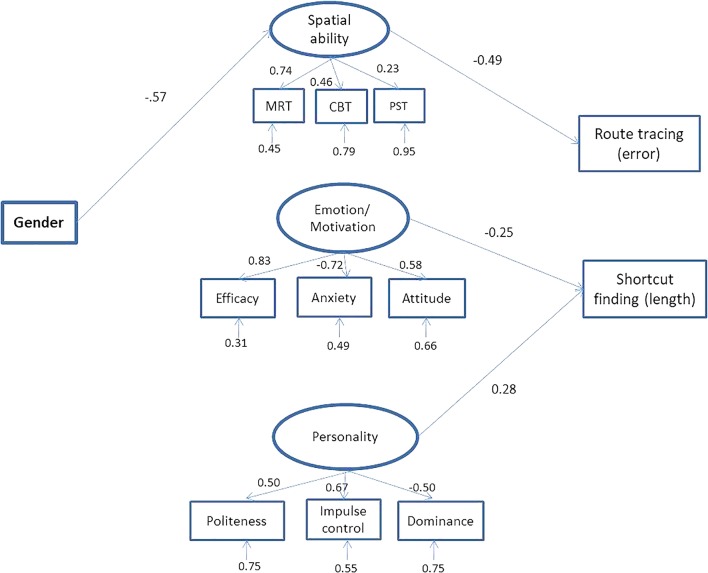
Final structural model. The standardized solutions (β) are presented for each path (all significant, *z* = –1.97 *p* < 0.05 to *z* = –0.3.76 *p* < 0.001).

In a first model, we considered all the relationships between the three latent variables and the two navigation tasks (route tracing and shortcut finding). The model showed satisfactory fit indices, χ^2^ = 46.64, *df* = 46 *p* = 0.45, *CFI* = 1.00, *NNFI* = 1.00, *SRMR* = 0.07, *RMSEA* = 0.01, but some relations were not significant (*z* = -0.68 to *z* = 1.44), i.e., gender and emotion/motivation; gender and personality; gender and route-tracing task, gender and shortcut-finding task; emotion/motivation and route tracing; personality and route tracing; route tracing and shortcut finding. We therefore tested a second model in which these relations were removed. The final model, shown in **Figure 3**, was satisfactory, χ^2^ = 54.88, *df* = 53 *p* = 0.40, *CFI* = 0.99, *NNFI* = 0.99, *SRMR* = 0.08, *RMSEA* = 0.02, and explained 24% of the variance for route tracing (*R^2^* = 0.24), and 14% of the variance for shortcut finding (*R^2^* = 0.14). Route-tracing performance was predicted by the spatial ability latent variable, which mediated the relationship between gender and route tracing (indirect effect: β = 0.28, *z* = 4.21 *p* ≤ 0.001). A different pattern emerged for shortcut finding, which was predicted by emotion/motivation and personality latent variables: high scores for self-efficacy and pleasure in exploring, and low scores for spatial anxiety were associated with a good performance. Personality predicted shortcut-finding performance too: low scores for Politeness (a facet of Agreeableness), and Impulse Control (a facet of Emotional Stability), and high scores for Dominance (a facet of Energy) were associated with a good performance.

## Discussion

Overall, the results of the study confirmed our expectations.

The preliminary analysis on participants’ performance in the two navigation tasks (route tracing and shortcut finding) revealed wide individual differences. These preliminary results confirmed previous reports of marked individual differences in performance in spatial navigation and orientation tasks (e.g., Hegarty and Waller, 2005; Hegarty et al., 2006; Weisberg et al., 2014; see also Iaria and Burles, 2016, on specific developmental deficits in topographical orientation).

The subsequent analyses aimed to test distinctive patterns of relationships between cognitive abilities, emotion/motivation, and personality traits on the one hand, and navigation task performance on the other. The correlation analyses showed that route tracing and shortcut finding related differently to the other variables: route-tracing performance correlated with the MRT and two VSWM tasks, which revealed no significant correlations with the shortcut-finding task; the latter task correlated instead with spatial anxiety and pleasure in exploring and self-efficacy. As for personality, it is worth noting that Perseverance (a facet of Conscientiousness), correlated with route-tracing performance, whereas it was Politeness and Impulse Control (facets of Agreeableness and Emotional Stability, respectively) that correlated significantly with shortcut-finding performance.

We also found interesting correlations between the predictive variables. As expected, the two VSWM tasks correlated with one another, and one of them (the Corsi Blocks Task) showed a moderate correlation with the MRT. This latter result supports the conviction that VSWM is implicated in the performance of figural spatial tasks (Allen et al., 1996; Hegarty et al., 2006; Muffato et al., 2016). All the measures of emotions and self-efficacy relating to spatial tasks showed reciprocal correlations: higher scores for spatial anxiety corresponded to lower scores for self-efficacy in spatial tasks and for pleasure in exploring. This supports the existence of reciprocal relationships between affective and motivational factors in the spatial performance domain, as already seen in other domains (e.g., Bandura, 1997). It also confirms and extends the report from Bryant (1982) of participants who admitted that they feared getting lost also reporting a lack of self-confidence. It is worth noting that spatial anxiety also correlated with some personality facets, suggesting a complex relationship between a general difficulty in controlling negative emotions (Emotion Control) and anxiety in spatial navigation tasks (on this point, see also Kallai et al., 2007). Interestingly, individuals with high levels of spatial anxiety were also less open to novel experiences, whereas no such relationship emerged between spatial anxiety and openness to culture.

Based on the above-described correlations, we tested a model grouping the variables into three latent factors: a spatial ability factor (grouping the MRT and the VSWM tasks), an emotion/motivation factor (with spatial anxiety, pleasure in exploring, and self-efficacy), and a personality factor (including the facets correlating the most with the shortcut-finding task). The model showed good fit indices and enabled us to test the predictive value of the three factors vis-à-vis route-tracing and shortcut-finding performance with a structural equation model. The main outcome of this last analysis was that performance in the two navigation tasks was predicted by a distinct order of variables. Spatial ability predicted route-tracing performance, confirming the results of previous studies showing that VSWM is implicated in navigation tasks (Allen et al., 1996; Garden et al., 2002; Meilinger et al., 2008; Labate et al., 2014), but not shortcut-finding performance. The latter result seems to contradict previous reports. For instance, Labate et al. (2014) found that a concurrent WM task impaired performance in shortcut-finding tasks in a real environment. This apparent discrepancy could be due to differences between the two studies: our study was conducted in a virtual outdoor urban environment with wide streets and numerous landmarks visible from a distance; the study by Labate et al. (2014) was conducted inside a real building on a university campus, with rooms connected by corridors and staircases, the routes to learn involved moving from one floor to another, and the landmarks were not visible from a distance. It may be that finding a shortcut in such an indoor environment demanded the ability to retain a mental representation of movements, locations of landmarks, and layouts of rooms, which would involve the use of VSWM. On the other hand, participants in our study could refer to landmarks some distance away to pinpoint their destination, and head toward it using navigation strategies that would be less demanding in terms of VSWM, but require a greater degree of confidence in participants’ ability to orient themselves, a positive attitude to exploring, and low levels of spatial anxiety. This view is also supported by our findings concerning the role of the personality latent factor comprising Politeness, Impulse Control, and Dominance in predicting shortcut-finding performance. In other words, an individual who is more likely to take the initiative (more dominant) and be impulsive (low impulse control), and less likely to consider other people’s requirements (less polite), is probably more inclined to embark on a totally new route, relying on a landmark in the distance. Taken together, all the above elements could explain why personality and emotional/motivational factors proved much more important than cognitive factors in explaining shortcut-finding performance. The route-tracing task, on the other hand, involved repeating a known route. To do so, participants needed to encode and maintain a sequential order of changes of direction and landmarks, and their spatial abilities (comprising VSWM and MRT) had a major part to play.

Some inconsistencies emerged when we compared our results with those of previous studies on the influence of personality traits on spatial task performance. Walkowiak et al. (2015) found high scores for psychoticism associated with a worse performance in a WF task, an outcome partially contradicted by our results, in which high scores for impulse control and politeness were associated with a worse performance in the shortcut-finding task. Here again, the difference is probably due to differences between the tasks involved. In the study by Walkowiak et al. (2015), participants had to retrace their steps, returning from the destination to the starting point of a previously memorized route, whereas our tasks involved repeating a route (going in the same direction as in the learning phase), and finding a shortcut. The environment used in the former study only allowed for participants to refer to local (not more remote) landmarks, and it was probably important for them to control their anxiety and fear of getting lost in order to reach their destination. In our route-tracing task, it was less important to control any negative emotions because participants traced the same route again [instead of going in the opposite direction, as in Walkowiak et al.’s (2015) study] and, more importantly, if they made three mistakes at the same intersection, they were told which way to go, so any fear of getting lost or spatial anxiety would naturally have been more limited. It would be interesting to manipulate such environmental features and procedural variables in the same study to clarify their influence on performance, and importance as predictive variables. To give an example, Pazzaglia et al. (2017) compared two conditions, with and without landmarks, in the same VE, and found that self-efficacy and pleasure in exploring became more important when the task was more difficult (in the no landmarks condition). Srinivas (2011) also found that spatial anxiety has a more harmful effect in difficult than in easy tasks.

## Conclusion

Overall, the results of the present study confirm that the type of environment, the type of task, and internal factors interact in contributing to WF performance (Pazzaglia and Meneghetti, 2017), but the whole picture is much more complex. Apparently trivial features of the task and environment can have a major impact, not only on performance, but also on the abilities required. A number of accurate classifications of WF tasks have been proposed in the past (e.g., Allen, 1999; Montello, 2005; Wiener et al., 2009), and proved very useful, but to understand the complex interaction between individual factors, environment and task, we probably need to draw finer distinctions. It is also important to bear in mind that, although many studies have examined individual differences in spatial navigation, they have focused largely on cognitive variables (WM, spatial ability). The present study underscores the importance of systematically considering other types of variable and referring to current models of emotions and their effect on cognition and motivation (Mischel and Shoda, 1995). How they affect the spatial domain needs to be further explored, also considering the same issues in samples at different level age (e.g., older than the age group considered in the presents research). The present study paves the way to research into how these factors influence performance in different WF tasks, and in the presence of different environmental features.

## Author Contributions

FP and CM conceived of the research and designed the experiment. CM and LR analyzed the data. FP drafted the manuscript. CM and LR provided the critical manuscript revisions.

## Conflict of Interest Statement

The authors declare that the research was conducted in the absence of any commercial or financial relationships that could be construed as a potential conflict of interest.
